# Herbal Medicine Processing By-Products as Bioactive Resources: In Vivo Evidence for Antioxidant, Anti-Inflammatory, and Immunomodulatory Effects

**DOI:** 10.3390/molecules31142516

**Published:** 2026-07-18

**Authors:** Ji Hye Hwang, Jin-Ho Jung

**Affiliations:** 1 Department of Acupuncture & Moxibustion Medicine, College of Korean Medicine, Gachon University, Seongnam 13120, Republic of Korea; 2 Namsangcheon Korean Medicine Clinic, Seoul 06656, Republic of Korea

**Keywords:** herbal medicine by-product, traditional Chinese medicine, Korean medicine, antioxidant, anti-inflammatory, immunomodulatory, in vivo

## Abstract

Processing herbal medicine generates substantial quantities of by-products that are discarded as waste. These residues may retain polysaccharides, flavonoids, saponins, terpenoids, and other constituents with pharmacological relevance. This review discusses the bioactive potential of by-products derived from single traditional Chinese and Korean herbal medicines, with emphasis on antioxidant, anti-inflammatory, and immunomodulatory mechanisms documented in preclinical in vivo studies. Representative in vivo evidence suggests that selected residues, marc fractions, fermentation products, and re-extracted by-products were reported to modulate oxidative stress markers, inflammatory cytokines, immune responses, and tissue-protective pathways across diverse experimental models. Processing method appears to be a key determinant of activity, as fermentation, high-temperature/high-pressure treatment, re-extraction, and targeted purification may enhance or diversify residual bioactivity. Current evidence remains limited by inconsistent chemical characterization, heterogeneous animal models, and incomplete reporting of experimental design. Re-evaluating herbal medicine processing by-products as bioactive resources may support sustainable herbal medicine manufacturing and circular bioeconomy frameworks.

## 1. Introduction

Traditional Chinese medicine (TCM) and Korean medicine (KM) have been integral to healthcare systems across East Asia for millennia, and a growing body of pharmacological evidence supports the biological activities of individual herbal constituents [1]. The global herbal medicine market has expanded substantially in recent decades, with large-scale industrial manufacturing of standardized herbal extracts, oral liquids, granules, and capsules representing a multi-billion-dollar sector [2]. However, this industrialization has correspondingly generated a significant and largely unaddressed problem: the accumulation of substantial quantities of processing by-products.

The by-products of herbal medicine processing—commonly referred to as herbal marc, spent herb, or drug residue—are solid or semi-solid materials that remain after the primary extraction of bioactive constituents from medicinal herbs. For every kilogram of finished herbal medicine product, an estimated 20–70% of the original plant material remains as processing residue [3,4,5,6]. In China alone, TCM processing generates residue exceeding tens of millions of tons annually [3,4,5,6], and a similar proportion of waste is generated in Korean red ginseng, deer antler, and other major KM industries [7].

Currently, the predominant practice is to discard these by-products as agricultural or industrial waste or, at best, to repurpose them as animal feed or compost [8]. This represents both an environmental burden and a significant economic loss. Primary extraction processes, particularly water decoction, are selective for water-soluble constituents but are less efficient at extracting lipophilic compounds, including terpenoids, flavonoid aglycones, and essential oil components [9]. The fermentation of herbal residues can generate novel bioactive metabolites through biotransformation [10,11]. Critically, emerging evidence has increasingly challenged the assumption that the by-products of herbal processing are pharmacologically inert after primary extraction.

Although this review focuses on herbal medicine processing by-products rather than conventional food-processing residues, many of the reviewed materials are plant-derived residues generated through extraction, decoction, fermentation, distillation, or related processing procedures. From the perspective of food chemistry, natural products research, and circular bioeconomy, these residues represent underutilized phytochemical resources with residual bioactive properties relevant to plant-derived by-product valorization.

Despite this mechanistic rationale, the pharmacological characterization of such by-products remains fragmented. To our knowledge, no previous review has specifically focused on the in vivo biological activities of by-products derived from single identifiable traditional herbal medicines using molecular-level biomarker evidence. This review aims to: (i) discuss the available in vivo evidence for the antioxidant, anti-inflammatory, and immunomodulatory activities of by-products derived from single herbal medicine processing; (ii) characterize by-product types, processing methods, and animal models; (iii) identify mechanistic findings and priority areas for future research.

## 2. Scope and Literature Basis

This review discusses representative in vivo studies concerning herbal medicine processing by-products. Relevant literature was identified through searches of PubMed, Embase, Web of Science Core Collection, Scopus, and CNKI. The final search was conducted on 30 April 2026, covering literature from January 2010 to April 2026. Grey literature was not systematically searched. Studies were selected to represent key categories of by-products, residual bioactive constituents, and biological activities across diverse animal models. The scope was restricted to by-products derived from single, identifiable herbal medicines to enable mechanistic attribution.

### 2.1. Scope of the Review

This review focused on in vivo studies that examined by-products generated during the processing of single, identifiable TCM or KM herbs. Specifically, the following scope was applied: (i) Population: Any animal model, including disease-induced and non-disease models; ex vivo studies were excluded. (ii) Intervention: By-products generated while processing a single, identifiable TCM or KM herb; multi-herb composite residues were excluded. (iii) Comparator: An appropriate control group. (iv) Outcomes: At least one molecular-level biomarker of antioxidant activity (superoxide dismutase (SOD), glutathione (GSH), malondialdehyde (MDA), reactive oxygen species (ROS), thiobarbituric acid reactive substances (TBARS)), anti-inflammatory activity (tumor necrosis factor (TNF)-alpha, interleukin (IL)-1 beta, IL-6, inducible nitric oxide synthase (iNOS), cyclooxygenase-2 (COX-2), NO), or immunomodulatory activity (IgG, IgA, IgE, IL-2, IL-4); studies reporting only gross physiological or behavioral endpoints without molecular biomarker data were excluded. (v) Study design: In vivo animal studies; ex vivo studies were excluded. (vi) Original peer-reviewed research in English, Korean, or Chinese, from January 2010 to April 2026.

### 2.2. Literature Basis

Five electronic databases were searched: PubMed, Embase, Web of Science Core Collection, Scopus, and China National Knowledge Infrastructure (CNKI) for Chinese-language literature. The search combined terms for by-products or residues of herbal medicine processing and biological activities, including antioxidant, anti-inflammatory, and immunomodulatory effects. In vivo animal studies were identified through title/abstract and full-text screening according to the scope of this review. For CNKI, additional filters were applied before deduplication to exclude composting, pesticide residues, and non-pharmacological agricultural applications.

### 2.3. Selection of Representative Evidence

Relevant records were screened at the title, abstract, and full-text levels to identify representative in vivo studies suitable for mechanistic discussion. When key information was not available in the full text, it was described as “not reported.”

### 2.4. Evidence Organization

The following information was summarized for each study: bibliographic information; herb name; by-product type and preparation method; animal model and disease induction; route, dose, and duration; control groups; quantitative outcome data with statistical significance; histopathological findings; and proposed mechanism of action. For studies including combined intervention groups (e.g., primary extract and residue), only data from single-herb residue groups were summarized and discussed in this review.

### 2.5. Methodological Features of the Available Evidence

Several methodological features were considered when interpreting the available in vivo evidence, including: (1) randomization; (2) allocation concealment; (3) baseline comparability; (4) housing conditions; (5) blinding of outcome assessment; (6) incomplete outcome data; and (7) selective outcome reporting. These features were used to guide interpretation of the strength and limitations of the available evidence.

### 2.6. Thematic Organization

Because the available studies used diverse herbal materials, by-product types, animal models, and outcome measures, this review organizes the evidence thematically by biological activity and mechanistic domain.

## 3. Results

### 3.1. Overview of Reviewed Evidence

**Figure 1:**
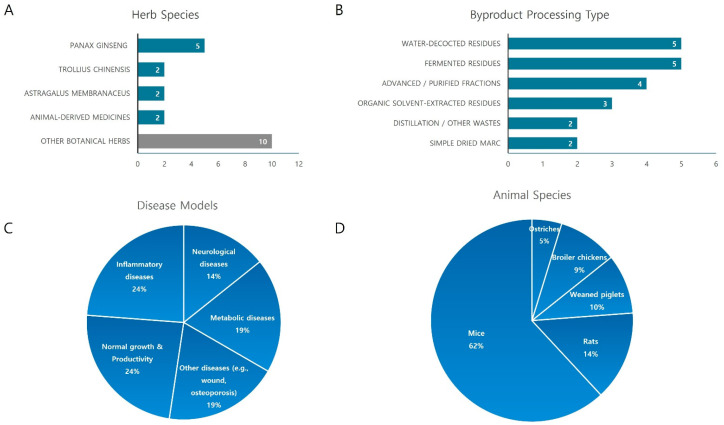
Distribution of characteristics among reviewed studies (*n* = 21). (**A**) Herb species. (**B**) By-product processing type. (**C**) Disease models. (**D**) Animal species. Other botanical herbs in panel A indicate ten single-study herbs each represented by one study.

The 21 reviewed studies [12,13,14,15,16,17,18,19,20,21,22,23,24,25,26,27,28,29,30,31,32] were published between 2014 and 2026 and encompassed 15 distinct herbal medicines. By-product sources included plant-derived residues (*n* = 19) and animal-derived residues (*n* = 2; deer antler and *Periplaneta americana*). By-product types included water-decocted marc or residue (*n* = 8), fermentation products of residues (*n* = 5), ethanol-extracted marc or residue (*n* = 4), essential oil by-products of steam distillation (*n* = 1), dried marc without re-extraction (*n* = 2), and purified protein fractions from residues (*n* = 1). Animal models comprised laboratory rodents (*n* = 14); livestock, including broiler chickens and piglets (*n* = 5); and African ostriches (*n* = 1). The predominant route of administration was oral gavage (*n* = 13), followed by dietary admixture (*n* = 5), topical application (*n* = 2), and intravaginal instillation (*n* = 1). Rodent disease models provided primary pharmacological evidence, whereas livestock studies mainly supported antioxidant, immunomodulatory, and intestinal health effects in applied dietary contexts. The detailed characteristics are presented in Table 1. The distribution of study characteristics by herb species, by-product type, disease model, and animal species is summarized in Figure 1.

### 3.2. Biological Activities

The biological activities of the reviewed by-products are organized into five thematic categories based on the primary disease models and outcome measures reported: (1) anti-inflammatory and antioxidant activity in local inflammation, atopic dermatitis, and wound healing; (2) neuroprotective activity; (3) metabolic regulatory activity; (4) organ protection and intestinal health; and (5) immunomodulatory, antioxidant, and bone-protective activity. A single study may appear across multiple categories when it reported outcomes relevant to more than one domain.

#### 3.2.1. Anti-Inflammatory and Antioxidant Activity in Local Inflammation, Atopic Dermatitis, and Wound Healing

Four studies demonstrated a statistically significant suppression of pro-inflammatory cytokines or oxidative stress markers in vivo. The ethyl acetate re-extract of *Sophora flavescens* residue (RSF; 200 mg/kg, oral, 5 days) inhibited xylene-induced auricular edema by 41.2%, acetic acid-induced peritoneal permeability by 50.2%, and carrageenan-induced hind paw edema by 72.0% at the 6 h time point (*p* < 0.01) [12]. Parallel in vitro experiments confirmed suppression of NO, TNF-α, IL-6, and monocyte chemoattractant protein-1 (MCP-1), implicating prostaglandin E2 (PGE2) pathway inhibition as a core mechanism.

The essential oil by-product of *Cinnamomum camphora* steam distillation (BEO; 70%, topical, 6 days) inhibited auricular edema by 72%, comparable to that of dexamethasone acetate [13]. Serum and tissue IL-1β, IL-6, and TNF-α were significantly downregulated at both protein and mRNA levels (*p* < 0.0001). Fermented red ginseng marc (fRGM; 100–400 mg/mL, topical, 6 weeks) significantly attenuated 2,4-dinitrochlorobenzene (DNCB)-induced atopic dermatitis, reducing serum IgE by 65.8–81.1% and suppressing Th2 cytokine mRNA expression [15]. Waste generated during production of deer antler oral liquid (ZX; 0.010 g/mL, topical, 9 days) promoted wound healing, with the strongest repair activity at low concentrations (*p* < 0.05), as confirmed by histopathological evidence of fibroblast proliferation and neovascularization [16].

Yeast-fermented *Trollius chinensis* residue (EGRF; 160 mg/kg, intravaginal, 7 days) significantly reduced vaginal fungal burden (colony-forming unit (CFU) score: 9.67 to 6.84, *p* < 0.01) with an 80% negative conversion rate in a murine candidal vaginitis model [17]. Additionally, yeast-fermented *Trollius chinensis* residue administered as a dietary supplement (4% admixture, 42 days) significantly increased serum total-SOD (T-SOD), GSH-peroxidase (Px), and total antioxidant capacity (T-AOC) (*p* < 0.05–0.01) and reduced malondialdehyde (MDA) in broiler chickens, while also significantly increasing thymus index, bursa of Fabricius index, and lymphocyte stimulation index (*p* < 0.01), demonstrating concurrent antioxidant and immunomodulatory activity [14].

#### 3.2.2. In Vivo Evidence of Neuroprotective Effects 

Two studies examined herbal by-products in neurological disease models, both focusing on Alzheimer’s disease. Korean red ginseng marc-derived gintonin (KRGM-G) (100 mg/kg, oral, 4 months) significantly improved spatial and reference memory in 5xFAD Alzheimer’s disease transgenic mice [21]. Amyloid-beta accumulation was markedly reduced. IL-1 beta, IL-6, TNF-alpha, COX-2, and iNOS in the frontal cortex were significantly decreased. Western blot revealed selective upregulation of lysophosphatidic acid receptor 1 (LPAR1) protein, suggesting neuroprotective effects via LPAR1-mediated Nrf2 antioxidant pathway activation and concurrent suppression of p38 mitogen-activated protein kinase (MAPK), nuclear factor kappa B (NF-κB) p65, and signal transducer and activator of transcription (STAT3) signaling.

*Curcuma longa* L. post-aqueous extraction residue (CLR; 1.2 g/kg, oral for 3 weeks) significantly improved spatial learning and memory in an Aβ1–42-induced Alzheimer’s disease mouse model [23]. CLR treatment significantly suppressed microglial overactivation, as evidenced by reduced Iba-1 expression (*p* < 0.01). Toll-like receptor 4 (TLR4) mRNA and protein levels and phosphorylation of IkappaB kinase (IKK), IkB, and p65 were significantly reduced (*p* < 0.01). Pro-inflammatory factors IL-6, TNF-alpha, iNOS, and COX-2 were decreased, while anti-inflammatory factors IL-4, IL-10, and TGF-beta 1 were increased. High-performance liquid chromatography (HPLC) analysis confirmed that CLR retained substantial curcuminoids (curcumin 16.5–19.94 mg/g), indicating that water-insoluble curcuminoids are preserved in the residue following aqueous extraction and are responsible for the observed TLR4/nuclear factor kappa B (NF-κB)-mediated neuroprotective effects. Together with the KRGM-G findings, these two studies demonstrate that by-products from herbal processing of distinct herbal sources can independently suppress the NF-κB neuroinflammatory pathway in Alzheimer’s disease models through different upstream targets (LPAR1 vs. TLR4).

#### 3.2.3. In Vivo Evidence of Metabolic Regulatory Effects 

Four studies investigated herbal processing by-products in metabolic or neuroendocrine-related regulatory contexts, including obesity, depression, and hyperlipidaemia. Notably, KRGM-G, which demonstrated neuroprotective effects in Section 3.2.2, also attenuated high-fat diet-induced obesity without altering food intake, upregulating brown adipose tissue (BAT) uncoupling protein 1 (UCP1) and hormone-sensitive lipase (HSL) mRNA while suppressing adipose TNF-alpha mRNA (*p* < 0.05) [22], suggesting that gintonin may exert pleiotropic effects through LPAR1-mediated signalling across both neurological and metabolic contexts. *Rehmannia glutinosa* residue water extract (RWE; 4 g/kg, oral, 7 days) significantly reduced immobility time in tail suspension and forced swim tests (*p* < 0.05), and reversed reserpine-induced hypothermia, implicating monoaminergic system modulation [18]. *Schisandra chinensis* seed residue (9% dietary, 10 days) reduced serum TG levels by 15% and increased HDL-C levels by 22% (*p* < 0.05) [20]. High-temperature/high-pressure (HTHP)-processed *Panax ginseng* residue polysaccharide (4.8 g/kg, 45 days) significantly reduced serum triglyceride (TG), total cholesterol, and low-density lipoprotein cholesterol (LDL-C) (*p* < 0.01) and increased HDL-C levels (*p* < 0.05) [19].

#### 3.2.4. In Vivo Evidence of Organ-Protective and Intestinal Effects 

*Periplaneta americana* residue water extract (WEoPa; 49.13 mg/kg, oral, 10 days) significantly reduced serum aspartate aminotransferase (AST) and alanine aminotransferase (ALT) in alcohol-induced and carbon tetrachloride (CCl_4_)-induced acute hepatic injury models (*p* < 0.05–0.01) and hepatic MDA (*p* < 0.05) [24]. In the chronic CCl_4_ rat model (at 75.78 mg/kg), AST and ALT levels were significantly reduced (*p* < 0.01) and the thymus index significantly increased (*p* < 0.05). *Brucea javanica* oil-extraction residue extract (DBF; 100 mg/kg, oral, 7 days) significantly attenuated dextran sulfate sodium (DSS)-induced ulcerative colitis, reducing colon TNF-alpha, IL-1 beta, and interferon (IFN)-gamma (*p* < 0.01), with histopathological confirmation [25]. Isatidis root residue (IRR; 1.0% dietary, 28 days) significantly reduced diarrhea in weaned piglets (*p* < 0.001), increased villus height, suppressed intestinal IL-6 and TNF-alpha mRNA (*p* < 0.05), and increased claudin-1 expression [27].

#### 3.2.5. In Vivo Evidence of Immunomodulatory, Antioxidant, and Bone-Protective Effects

*Epimedium* water-extraction residue (0.5 g/kg, oral, 8 weeks) significantly increased bone volume (BV), bone surface (BS), and trabecular number (Tb.N), and decreased trabecular thickness (Tb.Th) in ovariectomized osteoporotic Sprague-Dawley (SD) rats, with the residue group demonstrating superior improvement compared with that of an equivalent dose of primary water extract (*p* < 0.05) [30]. Although direct inflammatory or oxidative stress markers were not reported in this study, the osteogenic effects of *Epimedium* residue are mechanistically relevant to the immunomodulatory and antioxidant framework of this review, as oxidative stress and pro-inflammatory cytokines are established contributors to bone resorption and osteoporosis pathogenesis. The superior efficacy of the residue compared with the primary extract suggests that water-insoluble constituents retained in the processing by-product may contribute to osteogenic activity through pathways relevant to antioxidant and immune regulation. *Astragalus membranaceus* residue-derived Se-yeast (0.5 g/kg, oral, 4 weeks) significantly reduced fasting blood glucose, increased IgG and IL-2 levels (*p* < 0.05), and restored the islet structure in immunocompromised diabetic rats [29]. Red ginseng marc (3% dietary, 35 days) reduced broiler breast muscle TBARS (0.051–0.033 mg MA/kg; *p* < 0.001) and serum total cholesterol (TC), LDL-C, and TG levels (*p* < 0.05) [28].

*Astragalus membranaceus* dried marc (1% dietary, 80 days) significantly increased serum and hepatic SOD, GSH-Px, and catalase (CAT) activities, and decreased MDA in young African ostriches (*p* < 0.05), with concurrent reductions in serum TG and LDL-C and an increase in HDL-C (*p* < 0.05) [31]. *Lactobacillus plantarum*-fermented *Scutellaria baicalensis* residue (2 kg/t dietary, 28 days) significantly increased serum T-SOD by 40.52% and decreased MDA by 62.97% (*p* < 0.05), while significantly increasing IgG by 42.08% and IL-4 by 38.28% (*p* < 0.05) in weaned piglets [26]. Intestinal *Escherichia* coli was significantly reduced, and *Lactobacillus* was significantly increased (*p* < 0.05). *Dioscorea polystachya* residue protein (DP1; 0.9 mg/kg, 10 days) significantly reduced MDA, reactive oxygen species (ROS), and 8-hydroxy-2′-deoxyguanosine (8-OHdG), suppressed NLRP3 inflammasome components, and restored NO and cGMP via the phosphoinositide 3-kinase (PI3K)/protein kinase B (Akt)/endothelial NO synthase (eNOS) pathway [32].

### 3.3. Methodological Considerations

Several methodological limitations were consistently observed across the reviewed in vivo studies. Although most studies reported random group allocation, the specific randomization methods were rarely described in sufficient detail. Allocation concealment procedures were largely unreported, and pre-registered study protocols were absent. Baseline characteristics were comparable across groups in most studies; one study [19] did not explicitly confirm baseline body weight comparability. Outcome data were generally complete across all reviewed studies.

Blinding of outcome assessment was explicitly reported in only three studies. One study described histopathological evaluation by a blinded histopathologist [15], another reported that randomization and data handling were conducted blindly according to NIH workshop recommendations [21], and a third stated that grip strength assessment was performed under blinded conditions [22]. Reporting of housing conditions was inconsistent; one study explicitly noted that cage location was not rotated during the experiment, raising potential environmental bias concerns [22]. Overall, the available evidence should be interpreted as mechanistically suggestive rather than confirmatory, and further studies with improved reporting standards are needed.

### 3.4. Evidence Map

**Figure 2:**
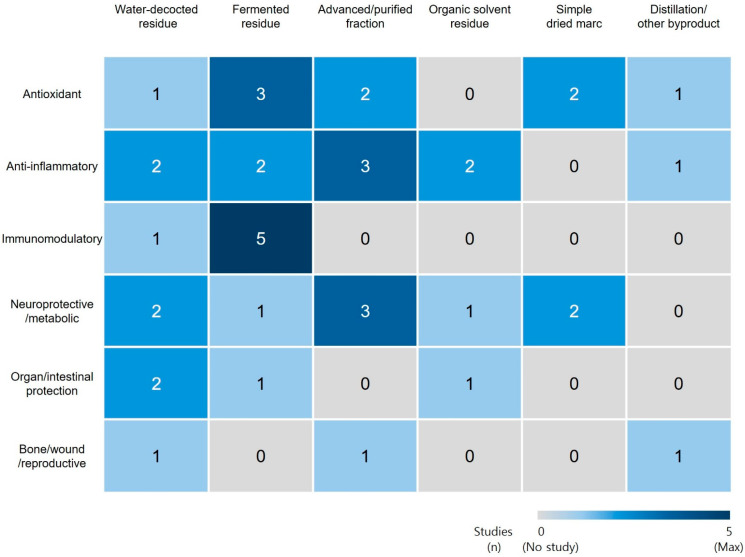
Heatmap-based evidence map showing the distribution of reviewed studies across by-product types and biological activity domains (*n* = 21). To construct this map, the reviewed studies were evaluated and their primary and secondary outcomes were categorized into six biological domains. By-products were classified into six types based on their specific extraction and processing methods. Cells indicate the number of reviewed studies mapped to each by-product type and biological activity category. Darker shading indicates a greater number of studies. Counts are not mutually exclusive because a single study may contribute to more than one biological activity category. The “distillation/other by-product” category includes steam distillation by-products and animal-derived processing residues.

A heatmap-based evidence map was generated to visualize the distribution of the reviewed studies across by-product types and biological activity domains (Figure 2, Table S1). Fermented residues showed the broadest activity coverage, particularly for immunomodulatory outcomes, whereas evidence for bone, wound, and reproductive outcomes was relatively sparse. A single study may contribute to multiple activity categories, in which multiple outcomes were reported.

## 4. Discussion

### 4.1. Summary of Main Findings

This review discussed evidence from 21 in vivo studies investigating the biological activities of by-products from single herbal medicine processing, derived from TCM and KM. The reviewed studies collectively suggest that selected by-products generated during herbal medicine manufacturing may retain measurable antioxidant, anti-inflammatory, and immunomodulatory activities across diverse disease models. These findings support a re-evaluation of the conventional assumption that these by-products are pharmacologically inert waste materials. Beyond their pharmacological significance, the valorization of the by-products of herbal medicine processing aligns with the principles of the circular bioeconomy and environmental sustainability [6,8,33,34], as large-scale herbal medicine manufacturing generates substantial quantities of residue that currently represent both economic and environmental burdens.

The included studies showed substantial heterogeneity in herbal species, by-product type, processing method, animal model, dose, intervention duration, and outcome measures (Table 2). Therefore, the available evidence was interpreted through a narrative synthesis rather than direct quantitative comparison.

### 4.2. Interpretation of Findings by Activity Category

#### 4.2.1. Anti-Inflammatory and Antioxidant Activity

A recurrent finding across several studies was the suppression of pro-inflammatory cytokines—particularly TNF-α, IL-1β, and IL-6—across diverse in vivo models. The RSF demonstrated broad anti-inflammatory activity across three independent acute inflammation models, with inhibition rates of 41.2%, 50.2%, and 72.0% [12]. In the reported model, BEO showed an edema-inhibitory effect comparable to that of dexamethasone acetate, accompanied by cytokine suppression at both the transcriptional and protein levels [13]. Fermented red ginseng marc modulated the Th1/Th2 balance through IgE (65.8–81.1%) and Th2 cytokine suppression, demonstrating that fermentation can generate enhanced and diverse biological activities [15]. The dual antioxidant and immunomodulatory activities of fermented *Trollius chinensis* residues in broiler chickens, which simultaneously improved antioxidant enzyme activities and immune organ development, further illustrate the capacity of fermentation processing to broaden the bioactivity profiles of by-products [14].

Across the reviewed studies, antioxidant and anti-inflammatory activities were frequently co-expressed rather than occurring in isolation. Reactive oxygen species (ROS) can activate pro-inflammatory transcription factors such as NF-κB, while anti-inflammatory signalling can in turn suppress oxidative stress pathways. This bidirectional relationship was evident in several reviewed studies: fermented *Trollius chinensis* residue simultaneously enhanced SOD and GSH-Px activities while upregulating immune organ indices [14], and fermented red ginseng marc attenuated both Th2-mediated inflammation and oxidative skin damage [15]. Understanding this antioxidant–anti-inflammatory axis is important for interpreting the mechanistic significance of the findings discussed in the sections that follow.

#### 4.2.2. Neuroprotective Activity

**Figure 3:**
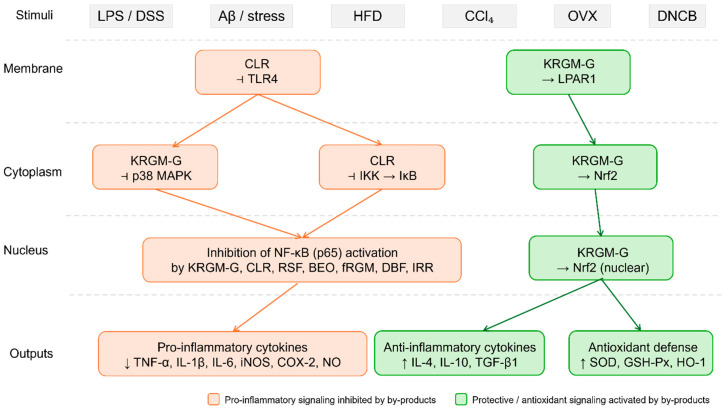
Proposed anti-inflammatory and antioxidant mechanisms of herbal medicine processing by-products. Representative by-products suppress NF-κB-related pro-inflammatory signaling and/or activate Nrf2-mediated antioxidant responses through upstream receptors and kinase cascades in response to diverse experimental stimuli. *Curcuma longa* residue (CLR) inhibits TLR4 activation and the IKK/IκB–NF-κB axis, whereas Korean red ginseng marc-derived gintonin (KRGM-G) modulates LPAR1/p38 MAPK signaling and activates the Nrf2 pathway. These changes were associated with decreased pro-inflammatory mediators and increased anti-inflammatory cytokines or antioxidant defense markers in the included studies. Arrowheads denote activation or signaling progression, T-shaped arrows denote inhibition, and ↑/↓ indicate increased/decreased expression or activity. Abbreviations: BEO, *Cinnamomum camphora* essential oil by-product; CLR, *Curcuma longa* post-aqueous extraction residue; DBF, *Brucea javanica* oil-extraction residue; fRGM, fermented red ginseng marc; IRR, *Isatis indigotica* root residue; KRGM-G, Korean red ginseng marc-derived gintonin; RSF, *Sophora flavescens* ethyl acetate residue extract.

Two studies examined herbal by-products in the context of Alzheimer’s disease, demonstrating convergent neuroprotective mechanisms through distinct upstream targets. KRGM-G demonstrated neuroprotective effects via selective LPAR1 upregulation, concurrent Nrf2/HO-1 activation, and p38 MAPK/NF-κB/STAT3 suppression in 5xFAD transgenic mice [21]. CLR demonstrated neuroprotective effects via TLR4/NF-κB pathway suppression and microglial overactivation inhibition in Abeta1–42-induced AD mice [23]. Although these studies utilized different herbal sources (red ginseng marc and turmeric residue), different disease models (5xFAD transgenic vs. Abeta1–42 injection), and different upstream targets (LPAR1 vs. TLR4), both converged on NF-κB pathway suppression as a central mechanism (Figure 3). This convergence across distinct by-product types supports NF-κB as a plausible shared mechanistic pathway in by-product-mediated neuroprotection. Notably, CLR retained substantial curcuminoids (16.5–19.94 mg/g) despite aqueous extraction, providing direct chemical evidence that lipophilic bioactives are preserved in herbal processing residues.

#### 4.2.3. Metabolic Regulatory Activity

Four studies investigated metabolic or neuroendocrine-related regulatory activities of herbal processing by-products, spanning obesity, depression, and hyperlipidaemia models. The thermogenic anti-obesity mechanism of KRGM-G via BAT UCP1 and HSL upregulation represents a potentially novel application as a high-volume manufacturing by-product [22]. The antidepressant-like activity of *Rehmannia glutinosa* RWE, despite markedly lower catalpol content (0.034 vs. 33.48 mg/g), indicates that other retained constituents contribute to monoaminergic system modulation [18]. Furthermore, the lipid-regulating effects reported for *Schisandra chinensis* seed residue [20] and HTHP-processed *Panax ginseng* residue [19] highlight their potential functional relevance in metabolic regulation, although translational applicability remains to be established.

#### 4.2.4. Organ Protection and Intestinal Health

The water extract of *Periplaneta americana* residue demonstrated broad-spectrum hepatoprotective activity across three distinct injury models, suggesting antioxidant-mediated hepatoprotection [24]. The efficacy of *Brucea javanica* oil-extraction residue in DSS-induced ulcerative colitis is consistent with that of retained quassinoid constituents following petroleum ether extraction [25]. Isatidis root residue has multifaceted intestinal health effects, simultaneously suppressing cytokines, upregulating tight junction proteins, and reducing *Campylobacter* counts [27]. The *Lactobacillus plantarum*-fermented *Scutellaria baicalensis* residue additionally demonstrated concurrent antioxidant and immunomodulatory effects in weaned piglets [26], further expanding the spectrum of intestinal health benefits achievable through herbal by-product supplementation.

#### 4.2.5. Immunomodulatory, Antioxidant, and Bone-Protective Activity

In one osteoporosis model, *Epimedium* water-extraction residue showed greater osteoprotective effects than an equivalent dose of the primary water extract, suggesting that lipophilic osteogenic flavonoids are preferentially retained in the residue because of their limited water solubility [30]. Although direct inflammatory biomarkers were not assessed in this study, the well-established relationship between oxidative stress and osteoporosis pathogenesis suggests that the observed bone-protective effects may be partially mediated through antioxidant and immunomodulatory pathways. Nevertheless, this interpretation remains speculative in the absence of direct biomarker evidence in the primary study. The antioxidant activity of Astragalus dregs in both the serum and liver of African ostriches demonstrated that the bioactive compounds retained after primary extraction maintained pharmacological relevance across diverse animal species [31]. The dual hypoglycemic and immunorestorative activities of Astragalus Se-yeast demonstrated the potential of fermentation technology for concentrating trace elements from herbal residues [29]. Similarly, dietary supplementation with red ginseng marc significantly improved lipid profiles and reduced oxidative stress (TBARS) in broiler chickens [28], reinforcing the broader applicability of herbal by-products as functional agricultural feed additives.

### 4.3. Role of Processing Method in Bioactivity Expression

Three distinct processing strategies were associated with enhanced or novel bioactivities. First, fermentation consistently expanded the bioactivity profile: five of the 21 reviewed studies employed fermentation of herbal residues. Yeast fermentation of *Trollius chinensis* residue generated antifungal activity not previously reported for the unfermented herb [17], microbial fermentation of red ginseng marc produced enhanced anti-allergic activity [15], *Lactobacillus plantarum* fermentation of *Scutellaria baicalensis* residue generated concurrent antioxidant and immune-enhancing activity [26], and Se-enriched yeast fermentation of Astragalus residue produced dual hypoglycemic and immunomodulatory effects [29]. Second, HTHP processing of *Panax ginseng* residue polysaccharides significantly enhanced the lipid-lowering efficacy through polysaccharide depolymerization [19]. Third, targeted purification—DP1 protein isolation [32] and KRGM-derived gintonin preparation [21,22]—yielded compounds with well-defined molecular targets and demonstrated specific biological activities: DP1 promoted erectile function recovery through penile tissue repair, while KRGM-derived gintonin exerted neuroprotective and anti-obesity effects through LPAR1-mediated signalling.

### 4.4. Strengths and Limitations

To the best of our knowledge, this is among the first reviews to focus specifically on the in vivo biological activities of by-products obtained from single herbal medicine processing, with the explicit exclusion of multi-herb composite residues. Methodological features of the available studies were considered when interpreting the evidence, including reporting of randomization, allocation concealment, blinded outcome assessment, housing conditions, incomplete outcome data, and selective outcome reporting. Blinded outcome assessment was explicitly described in three studies, and one study reported a limitation related to housing randomization. However, most studies lacked detailed descriptions of sequence generation and randomization procedures, indicating that the current evidence should be interpreted as mechanistically suggestive rather than confirmatory.

Several limitations should be acknowledged. First, although random allocation was explicitly stated in most studies, detailed methods of random sequence generation were rarely described; therefore, the certainty of the randomization procedures should be interpreted cautiously. Second, allocation concealment and outcome assessment blinding were insufficiently reported in most studies. Third, the marked heterogeneity of herbal materials, by-product types, models, and outcomes limited direct comparison across studies. Fourth, none of the reviewed studies reported pharmacokinetic data, limiting the assessment of clinical translatability. Fifth, by-product characterization and standardization were inconsistent across studies.

Beyond these methodological limitations, the translational relevance of the reviewed evidence is also constrained by several practical considerations. Industrial-scale recovery of bioactive compounds from herbal processing by-products presents challenges related to batch variability, extraction efficiency, and cost-effectiveness. Regulatory frameworks for by-product-derived materials vary across jurisdictions and remain underdeveloped in many countries, creating uncertainty around product classification and safety requirements. Furthermore, the lack of standardized quality control parameters for herbal by-products limits reproducibility and comparability across research settings. Addressing these translational barriers will require interdisciplinary collaboration across pharmacology, food science, regulatory science, and manufacturing engineering.

### 4.5. Implications for Future Research

This review identifies several priority areas for future research. First, the superior efficacy of the *Epimedium* residue warrants phytochemical profiling to identify its retained osteogenic constituents. Second, KRGM-derived gintonin and *Curcuma longa* residue warrant further preclinical validation, given their reported neuroprotective activities in Alzheimer’s disease models through distinct but convergent NF-κB-related mechanisms; clinical relevance remains to be established. Third, future studies on herbal medicine processing by-products should explicitly evaluate how processing conditions—including extraction solvent, temperature, duration, and fermentation parameters—affect the residual constituent profile and resulting biological activity. Comparative studies between crude residues and further-processed or purified fractions would help establish which processing steps are critical for bioactivity retention or enhancement. Additionally, future studies should adhere to the ARRIVE 2.0 guidelines [35] and incorporate prospective study protocols where feasible, blinded outcome assessments, randomized housing, and standardized by-product characterization.

Fourth, given the limited direct intestinal absorption of many residue-derived polysaccharides, future studies should investigate gut microbiota modulation as an indirect mechanism of bioactivity [36]—the gut microbiota–metabolism axis represents an important and currently underexplored pathway that warrants targeted investigation using 16S rRNA gene sequencing, shotgun metagenomics, and metabolomics approaches to elucidate systemic anti-inflammatory and immunomodulatory effects [37].

Fifth, the safety profile of the by-products of herbal processing requires rigorous evaluation before any translational or commercial application. Processing residues may accumulate heavy metals, pesticide residues, or mycotoxins at concentrations exceeding those of the primary herb [38,39], necessitating rigorous quality control frameworks [40]. Notably, one study conducted both acute and long-term toxicity evaluations of fermented *Scutellaria baicalensis* residues in Institute of Cancer Research (ICR) mice, demonstrating normal organ indices, liver function (AST/ALT within the reference range), and histopathological findings after 30 days of continuous administration, with no evidence of cumulative toxicity [26]. This represents one of the few reviewed studies to prospectively evaluate by-product safety, underscoring the feasibility of herbal by-product utilization when appropriately characterized and assessed. Future studies should incorporate standardized toxicological evaluations as an important component of by-product bioactivity research.

In addition, emerging methodological approaches offer promising avenues for advancing this field. Metabolomics-guided constituent profiling may accelerate the identification of bioactive residual compounds, while multi-omics integration (genomics, transcriptomics, and proteomics) could provide deeper mechanistic insights into by-product bioactivity. Artificial intelligence-assisted approaches, including machine learning-based prediction of bioactive constituents and network pharmacology, may further streamline the prioritisation of by-products with translational potential. Finally, future studies should integrate pharmacological evaluation with environmental and economic assessment, including life-cycle assessment (LCA)-based approaches, to clarify whether herbal by-product valorization can provide measurable benefits within circular bioeconomy, green pharmacy [41], and environmental, social, and governance (ESG) frameworks.

## 5. Conclusions

This review highlights the bioactive potential of herbal medicine processing by-products, with emphasis on in vivo evidence, molecular mechanisms, and sustainable valorization. Across the 21 reviewed studies encompassing 15 distinct herbal medicines, selected processing by-products showed measurable biological activities across a broad spectrum of disease models. Key findings include the retention of pharmacological activity attributable to residual bioactive constituents and processing method-dependent enhancement or diversification of bioactivity, particularly through fermentation. Notable examples include a greater osteoprotective effect of *Epimedium* residue than the primary water extract in one osteoporosis model, convergent NF-κB-related neuroprotective effects of KRGM-derived gintonin and *Curcuma longa* residue in Alzheimer’s disease models through distinct upstream targets, and one of the few prospective in vivo safety evaluations of a fermented herbal by-product through acute and long-term toxicity testing. Herbal medicine processing by-products represent a substantially underutilized resource, and their re-evaluation may offer a pathway toward value-added utilization aligned with circular bioeconomy principles and sustainable herbal medicine manufacturing.

## Figures and Tables

**Table 1:** Overview of In Vivo Studies Discussed in This Review.

Author/Year	Herb (Latin Name)	By-Product Type	Animal Model/Disease	Route/Dose/Duration	Key Outcomes	Histopathology	Mechanism
Ma et al. [12]	*Sophora flavescens*	EtOAc residue extract (RSF)	KM mice/Xylene oedema; acetic acid peritoneal; carrageenan paw models	Oral 200 mg/kg, 5 days	Auricular edema ↓ 41.2%; peritoneal permeability ↓ 50.2%; paw edema ↓ 72.0% (all *p* < 0.01)	—	PGE2 & NO/TNF-α/IL-6/MCP-1 suppression
Xiao et al. [13]	*Cinnamomum camphora*	Steam distillation by-product EO (BEO)	ICR mice/Xylene-induced auricular oedema	Topical 70% BEO, 6 days	Edema inhibition 72%; IL-1 beta, IL-6, TNF-alpha ↓ (protein & mRNA, *p* < 0.0001)	HE (ear tissue)	Suppression of inflammatory mediators at protein and mRNA levels
Ao et al. [14]	*Trollius chinensis*	Yeast-fermented residue	Broiler chickens (yellow-feathered)/Growth & antioxidant model	Dietary 2%, 4%, 6%, 42 days	T-SOD ↑, GSH-Px ↑, T-AOC ↑ (4% & 6%, *p* < 0.05–0.01); MDA ↓ (6%, *p* < 0.05); thymus index ↑, bursa index ↑, lymphocyte SI ↑ (4% & 6%, *p* < 0.01); survival rate ↑ (90–92%, *p* < 0.01)	—	Residual flavonoid-mediated antioxidant enzyme activation; immune organ development
Jung et al. [15]	*Panax ginseng*	Fermented red ginseng marc (fRGM)	SKH-1 mice/DNCB-induced atopic dermatitis	Topical 100–400 mg/mL, 6 weeks	GSH ↑, MDA ↓; IgE ↓ 65.8–81.1%; TNF-alpha, IL-4, IL-5, IL-13 mRNA ↓	HE, IHC (skin)	Antioxidant; Th2 cytokine & IgE suppression
Wei et al. [16]	*Cervus nippon*	Oral liquid production residue (ZX fraction)	KM mice/Skin wound model	Topical 0.010 g/mL, 9 days	Wound closure ↑ (*p* < 0.05); fibroblast proliferation & neovascularization; DPPH IC50 = 0.82 mg/mL	HE (skin)	Protein & polysaccharide-mediated tissue repair; antioxidant
Ji, Jiang & Han [17]	*Trollius chinensis*	Fermented residue extract (EGRF)	KM mice/*C. albicans* vaginitis	Intravaginal 160 mg/kg, 7 days	Vaginal fungal burden ↓ (CFU score: 9.67→6.84, *p* < 0.01); negative conversion 80%	HE (vaginal mucosa)	Direct antifungal; local anti-inflammatory
Wang et al. [18]	*Rehmannia glutinosa*	Residue water extract (RWE)	KM mice/Reserpine-induced depression	Oral, 2 & 4 g/kg, 7 days	TST & FST immobility time ↓ (*p* < 0.05, 4 g/kg); hypothermia reversed (*p* < 0.05)	—	Monoaminergic system modulation (putative)
Xie et al. [19]	*Panax ginseng*	HTHP residue polysaccharide	ICR mice/High-fat diet hyperlipidemia	Oral, 4.8 g/kg, 45 days	TG ↓↓, CHO ↓↓, LDL-C ↓ (*p* < 0.01); HDL-C ↑ (*p* < 0.05)	—	Polysaccharide depolymerization; lipid metabolism regulation
Chu et al. [20]	*Schisandra chinensis*	EtOH-extracted seed residue (FSC-SpEt)	ICR mice/HCBD diet hyperlipidemia	Dietary 9%, 10 days	Serum TG ↓ 15%, HDL ↑ 22% (*p* < 0.05); hepatic TC & TG ↓	—	Regulation of lipid metabolism & cholesterol absorption
Ha et al. [21]	*Panax ginseng*	KRGM-derived gintonin (KRGM-G)	5xFAD transgenic mice/Alzheimer’s disease	Oral 50 & 100 mg/kg, 4 months	Cognitive function restored; Abeta ↓; IL-1 beta, IL-6, TNF-alpha, COX-2, iNOS ↓; Nrf2 ↑, HO-1 ↑; LPAR1 ↑	HE, IHC, IF (brain)	LPAR1 upregulation → Nrf2/HO-1 activation & p38/NF-κB/STAT3 suppression
Yasmin et al. [22]	*Panax ginseng*	KRGM-derived gintonin	C57BL/6N mice/HFD-induced obesity	Oral 3x/week, 25 weeks	Body weight ↓; plasma TG & TC ↓; BAT UCP1 & HSL ↑; adipose TNF-alpha mRNA ↓ (*p* < 0.05)	HE, Oil Red O (liver, adipose)	Promotion of BAT thermogenesis; suppression of metabolic inflammation
Xu et al. [23]	*Curcuma longa*	Post-aqueous extraction residue (CLR)	C57BL/6 male mice/Abeta1–42-induced Alzheimer’s disease	Oral 1.2 g/kg, once daily, 3 weeks (CLR group only)	MWM escape latency ↓ (*p* < 0.01, day 4 onwards); Iba-1 (microglia) ↓ (*p* < 0.01); TLR4 mRNA & protein ↓; p-IKK, p-IkB, p-p65 ↓ (*p* < 0.01); IL-6, TNF-alpha, iNOS, COX-2 ↓; IL-4, IL-10, TGF-beta1 ↑; curcuminoids 16.5–19.94 mg/g confirmed in CLR	HE, Nissl, IHC, IF (brain)	Curcuminoid retention in CLR → TLR4/NF-κB pathway suppression → inhibition of microglial overactivation
Zhang et al. [24]	*Periplaneta americana*	Cold-maceration residue water extract (WEoPa)	KM mice & SD rats/CCl_4_, alcohol, APAP-induced hepatic injury	Oral 32.75–75.78 mg/kg, 10 days (acute); 7 weeks (chronic)	AST & ALT ↓ (*p* < 0.05–0.01); hepatic MDA ↓ (*p* < 0.05); thymus index ↑	—	Antioxidant hepatoprotection; immunomodulation
Gao et al. [25]	*Brucea javanica*	Oil-extraction residue EtOH extract (DBF)	BALB/c mice/3% DSS-induced ulcerative colitis	Oral 100 mg/kg, 7 days	DAI ↓; colon length preserved; TNF-alpha, IL-1 beta, IFN-gamma ↓ (*p* < 0.01)	HE (colon)	Pro-inflammatory cytokine suppression
An et al. [26]	*Scutellaria baicalensis*	*Lactobacillus plantarum*-fermented residue	Weaned piglets/Growth & immune model	Dietary 2 kg/t, 28 days	T-SOD ↑ 40.52% (*p* < 0.05); MDA ↓ 62.97% (*p* < 0.05); IgG ↑ 42.08%, IL-4 ↑ 38.28% (*p* < 0.05); *E. coli* ↓, *Lactobacillus* ↑; diarrhea ↓; safety confirmed (acute & chronic toxicity)	—	Biotransformation of L. *plantarum* fermentation; antioxidant enzyme activation; immune enhancement
Chen et al. [27]	*Isatis indigotica*	Water decoction residue (IRR)	Weaned piglets/Normal growth & intestinal health	Dietary 1.0%, 28 days	Diarrhea ↓ (*p* < 0.001); villus height ↑; IL-6 & TNF-alpha ↓ (*p* < 0.05); Claudin-1 ↑; *Campylobacter* ↓	HE, AB-PAS (intestine)	Intestinal barrier reinforcement; pro-inflammatory cytokine suppression
Kim, Lee & Choi [28]	*Panax ginseng*	Dried red ginseng marc (RGM)	Broiler chickens/Growth model	Dietary 1–3%, 35 days	TBARS ↓ (0.051→0.033 mg MA/kg, *p* < 0.001); TC, LDL, TG ↓ (*p* < 0.05)	—	Radical scavenging by Ginsenoside; inhibition of cholesterol absorption
Liu et al. [29]	*Astragalus membranaceus*	Se-enriched yeast fermentation product	SD rats/STZ+CP-induced diabetes & immunosuppression	Oral, 0.5 g/kg, 4 weeks	FBG ↓, INS ↑, WBC ↑, IgG ↑, IL-2 ↑ (*p* < 0.05); islet recovery	HE (pancreas)	Se-mediated antioxidant; immune restoration
Zhou et al. [30]	*Epimedium* spp.	Water-extraction residue (marc)	SD rats/OVX-induced osteoporosis	Oral 0.5 g/kg, 8 weeks	BV, BS, Tb.N ↑; 0.5 g/kg residue > water extract for BS, Tb.N, Tb.Th, Tb.Sp (*p* < 0.05)	Micro-CT (bone)	Residual flavonoid-mediated regulation of bone metabolism
Gong et al. [31]	*Astragalus membranaceus*	Dried residue marc (air-dried, 40-mesh powder)	African young ostriches/Growth & antioxidant model	Dietary 0.5–2%, 80 days	Serum & liver SOD ↑, GSH-Px ↑, CAT ↑, MDA ↓ (*p* < 0.05); TG ↓, LDL ↓, HDL ↑ (*p* < 0.05)	—	Residual polysaccharide & flavonoid; antioxidant enzyme activation; lipid metabolism
Yu et al. [32]	*Dioscorea polystachya*	Purified residue protein (DP1, ~12 kDa)	SD rats/Hydrocortisone-induced erectile dysfunction	Oral gavage, 0.3/0.6/0.9 mg/kg, 10 days	SOD ↑, MDA ↓, ROS ↓, 8-OHdG ↓; IL-6 & IL-1 beta ↓; NLRP3, ASC, Caspase-1 ↓; NO & cGMP ↑ (*p* < 0.01)	IF (penile tissue)	TXNIP/NLRP3 inflammasome inhibition; PI3K/Akt/eNOS pathway restoration

↓ indicates decrease; ↑ indicates increase; HE, haematoxylin and eosin; IHC, immunohistochemistry; IF, immunofluorescence; AB-PAS, Alcian Blue-Periodic Acid Schiff.

**Table 2:** Sources of heterogeneity across included studies (*n* = 21).

Domain	Categories Identified	Examples	Implication for Interpretation
Herbal species	15 distinct herbal medicines	*Sophora flavescens*, *Panax ginseng*, *Curcuma longa*, *Epimedium*, *Scutellaria baicalensis*, *Trollius chinensis*, and others	Different phytochemical profiles limit direct comparison across studies and restrict mechanistic attribution to individual herbal materials.
By-product type	Multiple processing-derived by-product categories	Water-decocted marc, fermented residue, ethanol-extracted marc, high-temperature/high-pressure (HTHP)-processed residue, and distillation by-product	Processing conditions may substantially alter residual constituent profiles and biological activity.
Experimental model	Multiple disease or functional models	Acute inflammation, atopic dermatitis, Alzheimer’s disease, obesity, hyperlipidaemia, hepatic injury, intestinal inflammation, wound healing, and bone loss	Outcomes are not directly comparable across disease contexts, limiting cross-study synthesis.
Animal species and strains	5 animal species, with multiple rodent strains	ICR, C57BL/6, and KM mice; Sprague-Dawley rats; broiler chickens; weaned piglets; and ostriches	Species- and strain-specific physiology and pharmacokinetics may limit cross-study generalisation.
Dose and administration route	Wide variation in dose and route	Oral administration, topical application, intravaginal administration, and dietary admixture; doses ranged from 0.3 mg/kg to 9% dietary supplementation	Dose–response relationships cannot be compared across studies.
Intervention duration	5 days to 25 weeks	Short-term acute models (e.g., 5 days for RSF) and longer-term chronic models (e.g., 4 months for KRGM-G in Alzheimer’s disease)	Short-term and long-term models reflect different biological contexts and are not directly comparable.
Outcome measures	Diverse molecular, histological, behavioural, and functional endpoints	Cytokine levels, antioxidant enzymes, histopathology, behavioural tests, bone morphometry, lipid profiles, and intestinal barrier-related markers	No common primary outcome was available, making quantitative synthesis inappropriate.
Chemical characterisation	Inconsistent depth of constituent analysis	Some studies reported HPLC profiling or relatively defined fractions/compounds (e.g., *Curcuma longa* residue curcuminoids, gintonin, DP1 protein, polysaccharide fractions); many studies used crude extracts without quantitative standardisation	Active constituents and quality-control standards remain insufficiently defined in many studies.

Abbreviations: DP1, deer antler residue-derived protein 1; HTHP, high-temperature/high-pressure; HPLC, high-performance liquid chromatography; ICR, Institute of Cancer Research; KM, Kunming; KRGM-G, Korean red ginseng marc-derived gintonin; RSF, *Sophora flavescens* residue extract.

## Data Availability

No new data were created or analyzed in this study. Data sharing is not applicable to this article.

## Supplementary Materials

The following supporting information can be downloaded at: https://www.mdpi.com/article/10.3390/molecules31142516/s1, Table S1: Mapping of reviewed studies to evidence map categories.

## Abbreviations

The following abbreviations are used in this manuscript:

TCM	Traditional Chinese medicine
KM	Korean medicine
SOD	superoxide dismutase
GSH	glutathione
MDA	malondialdehyde
ROS	reactive oxygen species
TBARS	thiobarbituric acid reactive substances
TNF	tumor necrosis factor
IL	interleukin
iNOS	inducible nitric oxide synthase
COX-2	cyclooxygenase-2
MCP-1	monocyte chemoattractant protein-1
PGE2	prostaglandin E2
CNKI	China National Knowledge Infrastructure
fRGM	fermented red ginseng marc
DNCB	2,4-dinitrochlorobenzene
T-AOC	total antioxidant capacity
KRGM-G	Korean Red Ginseng Marc-derived Gintonin
LPAR1	lysophosphatidic acid receptor 1
NF-κB	nuclear factor kappa B
TLR4	toll-like receptor 4
ESG	environmental, social, and governance
MAPK	mitogen-activated protein kinase
IKK	IkappaB kinase
HPLC	high-performance liquid chromatography
BAT	brown adipose tissue
UCP1	uncoupling protein 1
HSL	hormone-sensitive lipase
AST	serum aspartate aminotransferase
ALT	alanine aminotransferase
CCl_4_	carbon tetrachloride
BV	bone volume
BS	bone surface
Tb.N	trabecular number
Tb.Th	trabecular thickness
SD	Sprague-Dawley
PI3K	phosphoinositide 3-kinase
Akt	protein kinase B
eNOS	endothelial NO synthase
HTHP	high-temperature/high-pressure
ICR	Institute of Cancer Research
