# Supplementary material for: Herbal Medicine Processing By-Products as Bioactive Resources: In Vivo Evidence for Antioxidant, Anti-Inflammatory, and Immunomodulatory Effects
Source: Molecules. 2026 Jul 18;31(14):2516. doi: 10.3390/molecules31142516 (PMC13416048; doi:10.3390/molecules31142516)
Supplement: Supplementary file 1 [file molecules-31-02516-s001.zip › molecules-4373218-supplementary.pdf]

Supplementary Table S1. Mapping of reviewed studies to evidence map categories.

Study	Antioxidant	Anti-inflammatory	Immuno-modulatory	Neuroprotective / Metabolic	Organ / Intestinal protection	Bone / Wound / Reproductive
Ma et al. [12] (Sophora)	—	✓	—	—	—	—
Xiao et al. [13] (Cinnamomum)	—	✓	—	—	—	—
Ao et al. [14] (Trollius/fermented)	✓	—	✓	—	—	—
Jung et al. [15] (fRGM)	✓	✓	✓	—	—	—
Wei et al. [16] (*Cervus nippon*)	✓	—	—	—	—	✓
Ji, Jiang & Han [17] (Trollius/fermented)	—	✓	✓	—	—	—
Wang et al. [18] (Rehmannia)	—	—	—	✓	—	—
Xie et al. [19] (Panax/HTHP)	—	—	—	✓	—	—
Chu et al. [20] (Schisandra)	—	—	—	✓	—	—
Ha et al. [21] (KRGM-G/AD)	✓	✓	—	✓	—	—
Yasmin et al. [22] (KRGM-G/obesity)	—	✓	—	✓	—	—
Xu et al. [23] (Curcuma/AD)	—	✓	—	✓	—	—
Zhang et al. [24] (Periplaneta)	✓	—	—	—	✓	—
Gao et al. [25] (Brucea)	—	✓	—	—	✓	—
An et al. [26] (Scutellaria/fermented)	✓	—	✓	—	✓	—
Chen et al. [27] (Isatis)	—	✓	✓	—	✓	—
Kim, Lee & Choi [28] (Red ginseng marc)	✓	—	—	✓	—	—
Liu et al. [29] (Astragalus Se-yeast)	—	—	✓	✓	—	—
Zhou et al. [30] (*Epimedium*)	—	—	—	—	—	✓
Gong et al. [31] (Astragalus/ostrich)	✓	—	—	✓	—	—
Yu et al. [32] (Dioscorea)	✓	✓	—	—	—	✓
Total (n)	9	10	6	9	4	3

✓ = outcome reported; — = not reported. fRGM, fermented red ginseng marc; KRGM-G, Korean red ginseng marc-derived gintonin; HTHP, high temperature and high pressure; AD, Alzheimer's disease. Counts are not mutually exclusive; a single study may contribute to more than one category.
